# Food Web Architecture and Basal Resources Interact to Determine Biomass and Stoichiometric Cascades along a Benthic Food Web

**DOI:** 10.1371/journal.pone.0022205

**Published:** 2011-07-18

**Authors:** Rafael D. Guariento, Luciana S. Carneiro, Adriano Caliman, João J. F. Leal, Reinaldo L. Bozelli, Francisco A. Esteves

**Affiliations:** 1 Departamento de Ecologia, Universidade Federal do Rio de Janeiro, Rio de Janeiro, Rio de Janeiro, Brasil; 2 Centro Federal de Educação Tecnológica de Química, Nilópolis, Rio de Janeiro, Brasil; 3 Núcleo em Ecologia e Desenvolvimento Sócio-Ambiental de Macaé, Macaé, Rio de Janeiro, Brasil; 4 Departamento de Botânica, Ecologia e Zoologia, Universidade Federal do Rio Grande do Norte, Natal, Rio Grande do Norte, Brasil; Dalhousie University, Canada

## Abstract

Understanding the effects of predators and resources on primary producers has been a major focus of interest in ecology. Within this context, the trophic cascade concept especially concerning the pelagic zone of lakes has been the focus of the majority of these studies. However, littoral food webs could be especially interesting because base trophic levels may be strongly regulated by consumers and prone to be light limited. In this study, the availability of nutrients and light and the presence of an omnivorous fish (*Hyphessobrycon bifasciatus*) were manipulated in enclosures placed in a humic coastal lagoon (Cabiúnas Lagoon, Macaé – RJ) to evaluate the individual and interactive effects of resource availability (nutrients and light) and food web configuration on the biomass and stoichiometry of periphyton and benthic grazers. Our findings suggest that light and nutrients interact to determine periphyton biomass and stoichiometry, which propagates to the consumer level. We observed a positive effect of the availability of nutrients on periphytic biomass and grazers' biomass, as well as a reduction of periphytic C∶N∶P ratios and an increase of grazers' N and P content. Low light availability constrained the propagation of nutrient effects on periphyton biomass and induced higher periphytic C∶N∶P ratios. The effects of fish presence strongly interacted with resource availability. In general, a positive effect of fish presence was observed for the total biomass of periphyton and grazer's biomass, especially with high resource availability, but the opposite was found for periphytic autotrophic biomass. Fish also had a significant effect on periphyton stoichiometry, but no effect was observed on grazers' stoichiometric ratios. In summary, we observed that the indirect effect of fish predation on periphyton biomass might be dependent on multiple resources and periphyton nutrient stoichiometric variation can affect consumers' stoichiometry.

## Introduction

The interaction between consumers, producers and nutrients has been heavily explored and has become a central subject in ecological studies [1], [2]. However, the predictions and generalizations of consumer-producer interactions have mostly been based on studies of the pelagic habitat, not accounting for the fact that a substantial portion of the resources supporting top consumers can be generated in the benthic habitat [3]. Studies evaluating the trophic cascade effect in benthic habitats have shown that fish predation leads to a decrease in the number of herbivores or affects their foraging behavior, resulting in a positive indirect effect on primary producers (i.e., periphytic biomass) [4], [5], [6]. However, this effect has received mixed support throughout the literature [7]. In general, the strength of this cascade effect is dependent on resource availability [8], which is in agreement with the observed pattern for pelagic [9] and benthic [10] communities. Although the interaction between benthic organisms can take place in isolation, fish foraging activity can promote the coupling of different habitats, especially in shallow ecosystems. Fish are very mobile organisms that can actively participate in benthic and pelagic food webs [11], which could have potential implications in the strength of trophic cascades [12]. Additionally, although light penetration is controlled by phytoplankton shading in many lakes as a result of increased nutrient supply [13], most shallow and coastal aquatic ecosystems worldwide have low nutrient concentrations and low phytoplankton biomass, and the variation in light penetration to the benthic habitat is mainly controlled by variation in the input of colored terrestrial organic matter [14]. These characteristics makes these systems particularly prone to light limitation under low nutrient conditions.

The supply of light and nutrients not only affects the productivity of aquatic ecosystems [14], but may also have a strong effect on the ratio of elements in primary producers, which results in changes in their nutritional quality [15]. The stoichiometric “light∶nutrient hypothesis” states that ecosystems receiving large amounts of light relative to nutrients tend to yield nutrient-poor producers (resulting in high tissue C∶P or C∶N ratios), a theory that has been recently been applied to periphyton communities [16], [17], [18]. However, systems receiving low light∶nutrient supply ratios should yield more nutrient-rich producers that subsequently govern fundamental ecosystem processes, such as the transfer and allocation of energy among trophic levels [19]. Although producers may exhibit strong variability in nutrient composition [20], animals are believed to be more homeostatic. As a result of this perceived strict homeostasis, the interest in body nutrients imbalances of higher trophic levels has been fairly sparse, and relatively little is known about how changes in nutrient stoichiometry travel up the food chain [21]. However, empirical studies and a recent meta-analysis have shown that consumers are not strictly homeostatic, and physiological constraints may be a mechanistic explanation for consumers' body elemental flexibility [22], [23]. In addition, consumer-driven nutrient recycling can significantly affect primary producers [24]. Therefore, top-down and bottom-up food web concepts may not be exclusively concerned with trophic levels' biomass, and due to strong reciprocal influences of consumers and resources [25], “stoichiometric cascades” may be widespread.

Through a manipulative field experiment in aquatic enclosures, this study aimed to evaluate the individual and interactive effects of nutrients, light and an omnivorous fish on the biomass and nutrient stoichiometry of the periphytic community and its consumers in an oligo-mesotrophic humic coastal lagoon, which is a dominant aquatic ecosystem type in the Neotropical region [26]. We aimed to test the following hypotheses: (1) Fish predation on benthic herbivores will positively affect periphyton biomass, and these effects will be greater in high nutrient and light conditions; (2) based on the light∶nutrient hypothesis, lower light intensity will lead to lower periphyton C∶N and C∶P ratios; and (3) lower periphyton C∶N and C∶P ratios will lead to nutrient-richer consumer body tissues.

## Methods

### Experimental Site

This study was conducted in Cabiúnas Lagoon, located at Restinga de Jurubatiba National Park, Rio de Janeiro, Brazil (22°15′S, 41°40′W). This lagoon has a surface area of 0.35 km^2^ and a mean depth of 2.0 m. The water is humic (10–36 mg/L dissolved organic carbon, DOC) and slightly acidic (pH 6.3), with an average annual temperature of 23.6°C. During the study period, mean total phosphorus (TP) and nitrogen (TN) of the water were 1.5 µmol/L and 20 µmol/L respectively. Mean phytoplankton biomass estimated by chlorophyll-*a* (Chl-*a*) was 30 µg/L, and mean Secchi disk depth was 0.6 m. This lagoon is colonized by many species of freshwater snails, including *Biomphalaria tenagophila* (Gastropoda), which feed on periphyton as a main component of their diet [27].

### Experimental design

The experiment was conducted over a seven-week period using cylindrical transparent plastic containers of 2.0 m in diameter and 2.4 m tall. The experimental framework followed a 2×2 orthogonal whole-factor combination (+/− nutrients and +/− fish) with two levels (high and low) of a subplot-like factor (i.e., light) represented by two depths (Figure S1). This type of light manipulation mimics the natural variation in light availability in the system and has been used to evaluate light effects on periphyton biomass and stoichiometry in previous studies [28]. Whole-factor treatments were replicated four times in each possible combination: N0F0 (no fish or nutrient addition); N0F1 (only fish addition); N1F0 (only nutrient addition); N1F1 (both fish and nutrient addition), resulting in 16 enclosures. To evaluate potential influences of spatial heterogeneity, whole-factor treatments were distributed into four spatial block designs.

### Experimental Setup

In the treatments with nutrient addition, final concentrations of inorganic forms of nitrogen (N) and phosphorus (P) were kept constant throughout the experiment at 50 µM of N (adjusted with NH_4_NO_3_) and 10 µM of P (adjusted with KH_2_PO_4_ and K_2_HPO_4_). To maintain these concentrations, inorganic nutrient concentrations were assessed and adjusted weekly to the proper concentrations based on experimental standards. Fish density was manipulated by introducing 40 adult individuals of *Hyphessobrycon bifasciatus*, Ellis 1911, (Characidae) into the enclosures, achieving a final density of 13 ind./m^2^, which is similar to that found in the littoral region of the Cabiúnas lagoon [29]. Individual fish varied in size from 3 to 3.5 cm and biomass from 0.3 to 0.5 g/ind. *H. bifasciatus* is an omnivorous fish that forages on both pelagic and benthic food webs and can feed on zooplankton, phytoplankton, periphyton and detritus. Periphyton and juvenile Planorbid molluscs are important components of their diet, but molluscs are no longer susceptible to H. *bifasciatus* predation after they achieve a specific size due to gape limitation. *H. bifasciatus* was used in this experiment due to its ubiquity in many South American freshwater ecosystems [30] and broad foraging distribution throughout the water column. Light incidence was manipulated by incubating periphyton substrates (0.04 m^2^ plastic tiles) at two different depths (0.4 m (high light) and 1.4 m (low light)). The mean light stratification ranged from 40 to 400 µW/cm^2^ at the bottom and surface of the experimental enclosures at noon, respectively, and was established as statistically significant between each other at the experiment onset (*t*-test: *P*<0.05, *n* = 16). Water temperature and dissolved oxygen concentration were quite similar and constant at the bottom and top depths of the enclosures over the course of the experiment. Mean values of water temperature were 20.2 and 22.7°C at the bottom and top of the enclosures, respectively. In the same manner, dissolved oxygen concentrations were 7.2 and 8.5 mg/l, respectively. Therefore, light was the main abiotic variable co-varying with depth that could directly or indirectly influence the benthic community in this experiment. To encompass the greatest possible spatial heterogeneity in periphyton distribution, plastic tiles (0.015 m^2^) representing periphyton substrates were placed 1.0 m from each other in a radial arrangement along the enclosure wall 0.4 m and 1.4 m below the water's surface.

### Sampling and analysis

Depth-integrated water samples were collected weekly from each enclosure for assessment of pelagic Chl-*a* (used here as surrogate of phytoplankton biomass), total nitrogen (TN), total phosphorus (TP), dissolved inorganic and total forms of nitrogen (DIN and TDN) and phosphorus (DIP and TDP) concentrations. Nitrogen was measured after persulfate oxidation and nitrate reduction in a cadmium column with post-nitrite determination [31] except for ammonia measurements, which were obtained according to Solorzano [32]. Phosphorus was measured using the ammonium-molybdate method after persulfate oxidation [33]. Water transparency was measured as Secchi disk depth. Periphytic substrates were sampled weekly, conditioned into plastic containers filled with deionized water and immediately transferred to the laboratory. The tiles were scraped using a razor blade and the slurry was adjusted to a defined volume. The total periphyton biomass, periphyton chlorophyll-a (Chl-*a*) and nutrients were determined from the slurry. Total periphyton biomass was estimated as ash-free dry weight (AFDW). Samples were filtered in a pre-combusted 0.75-µm-pore filter and AFDW was determined after drying the filters (24 h at 70°C) for weight determination. Samples were then burned (2 h at 500°C) for AFDW determination. Periphyton Chl-*a* was estimated using the spectrofluorimetric method (Turner® 3A). Periphyton phosphorus (P) was measured according to Mackereth et al. (1978) and nitrogen (N) according to APHA (1989). Periphyton carbon (C) was measured using a carbon analyzer (TOC 5000 Shimadzu®) for solid samples after filtration in a pre-combusted 0.75-µm-pore filter.

At the experiment conclusion, all macro-invertebrates associated with the enclosure wall were collected by dragging a net (0.5-mm mesh size) along the enclosure wall and transferred to the laboratory. These individuals mostly comprised planorbidae Mollusca of the specimen *Biomphalaria tenagophila*, d'Orbigny 1835, and nearly all individuals were located on the upper part of the enclosure wall (Figure S2). We were unable to estimate the initial densities of these individuals for this experiment. However, such species are homogeneously distributed and commonly found on the sediment of the chosen experimental site. Additionally, because the analysis of the block effect on gastropod biomass at the experiment conclusion was not significant (see below), we concluded that treatment effects drove the observed final densities. Animals were dried for two days in an oven (70°C) and re-weighed to the nearest 0.01 mg to obtain the dry mass of the organisms. They were then ground into a coarse powder with a mortar and pestle. Powders were stored in glass vials for subsequent analysis of tissue C, N and P. To avoid any confounding effects, only individuals of the same size among all treatments were used in the analysis. Powder samples were analyzed for total N and P. Carbon analysis was performed using a Total Carbon Analyzer (TOC-5000 Shimadzu®).

### Statistical analysis

We used a repeated measures analysis of variance (RM-MANOVA) to evaluate the individual and interactive effects of time, fish, nutrients and light on total and autotrophic periphytic biomass, C∶Chl-*a* ratio and C∶N∶P ratios. Because this model was highly significant for both factorial and one-way designs, we performed separate univariate ANOVAs for each response variable. Periphyton biomass measurements and stoichiometry were analyzed in independent models. Biomass and stoichiometric measurements throughout the weeks were treated as multiple dependent variables measuring different levels of the repeated factors of time (*n* = 6) and light (*n* = 2). Light treatment levels were also treated as a repeated factor due to the spatial dependence of the experimental units [28], [34]. Independent factors in the model comprised fish (*n* = 2, present and absent) and nutrients (n = 2, with and without addition). Corrections due to the violation of the sphericity assumption were conducted using the Huynh-Feldt adjustment to the degrees of freedom. To reduce the heterogeneity of the variances, data were also log transformed the data. Contrast-Analysis (CA) was conducted as a post-test to evaluate the differences between treatment levels.

To evaluate statistical differences among treatments for pelagic concentrations of Chl-*a*, TN, TP, DIN, TDN, DIP, and water column transparency, we used independent repeated measures ANOVA (RM-ANOVA). Nutrients and fish were treated as categorical variables and time as the repeated factor. To evaluate snails' biomass and stoichiometric differences we used a two-way ANOVA with the HSD Tukey as a post-hoc test.

For all statistical models, a previous analysis was conducted including the experimental blocks (n = 4) as a categorical factor. Because no significant effect and negligible residual explicability were observed, experimental blocks were removed from the main analysis.

## Results

### Effects and efficiency of experimental manipulations

Phytoplankton biomass (Chl-*a*) was significantly higher in treatments with nutrient addition, especially in combination with fish presence (Table 1). The higher phytoplankton biomass in these treatments significantly reduced water transparency, detected as a reduction in Secchi disc depth (Table 1). Both TN and TP concentrations were significantly higher in the treatments with nutrient addition (Table 1), and dissolved forms followed the same pattern. Nutrient addition changed the N∶P ratio in the water to nearly 5∶1. We did not detect any effect of fish presence on average water nutrient concentrations (Table 1), overall or in a specific week (*P*>0.05 for the *Fish × Time* interaction for all nutrient forms).

**Table 1:** General comparison of the biotic and abiotic variables of the water column among treatments.

	Control	Fish	Nutrient	Fish + Nutrient
Pelagic Chl-*a* (µg.L^−1^)	7.9^a^ (1.2)	11.1^a^ (2.0)	19.7^a^ (7.5)	64.9^b^ (37.3)
Secchi disc (m)	1.0^a^ (0.3)	1.0^a^ (0.1)	0.96^a^ (0.1)	0.74^b^ (0.05)
DIN (µmol.L^−1^)	3.6^a^ (4.1)	3.9^a^ (1.2)	30.8^b^ (3.5)	24.7^b^ (5.5)
TDN (µmol.L^−1^)	5.6^a^ (21.9)	6.6^a^ (20.1)	39.2^b^ (76.0)	40.5^b^ (80.0)
DIP (µmol.L^−1^)	0.1^a^ (0.1)	0.1^a^ (0.1)	5.9^b^ (0.8)	6.1.^b^ (1.5)
TDP (µmol.L^−1^)	0.5^a^ (0.1)	0.5^a^ (0.2)	6.1^b^ (1.0)	6.3.^b^ (0.9)
TN (µmol.L^−1^)	13.95^a^ (1.8)	14.75^a^ (1.2)	49.32^b^ (2.5)	50.87^b^ (4.7)
TP (µmol.L^−1^)	0.8^a^ (0.2)	0.8^a^ (0.2)	8.65^b^ (1.0)	9.4^b^ (0.6)
TN∶TP	17.43^a^ (2.7)	18.43^a^ (4.4)	5.7^b^ (1.3)	5.4^b^ (0.9)

DIN = Dissolved inorganic nitrogen; TDN = Total dissolved nitrogen; DIP = Dissolved inorganic phosphorus; TDP = Total dissolved phosphorus; TN = Total nitrogen; TP = Total phosphorus.

Results are the mean (± SD) throughout the weeks (2nd to 7th week). Different letters above mean values represent significant differences (RM-ANOVA with Contrast Analysis as post hoc test, *P*<0.05).

### Biomass and nutrient content of benthic snails

**Figure 1:**
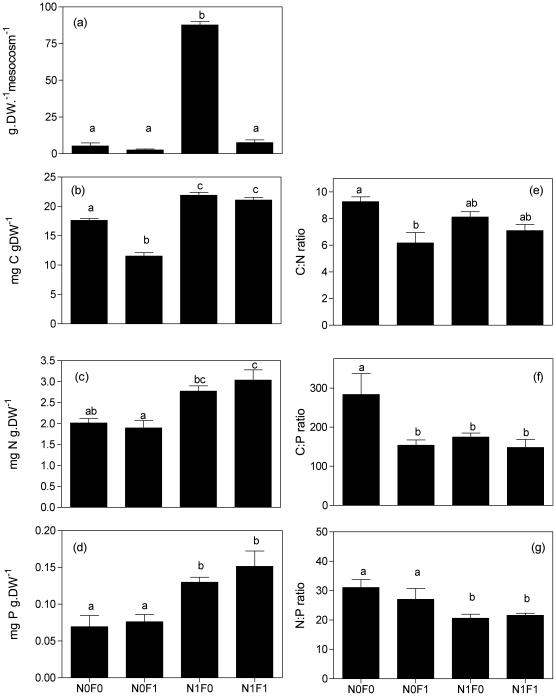
Biomass (a), Carbon content (b), Nitrogen content (c), Phosphorus content (d) and C∶N (e), C∶P (f) and N∶P (g) molar ratios of *Biomphalaria tenagophila* (Gastropoda). Treatments are represented by different nomenclatures (N0F0 (no fish or nutrients addition); N0F1 (only fish addition); N1F0 (only nutrients [N and P] addition); N1F1 (both nutrients and fish addition). Each bar represents mean values +1SE. Different letters above bars represent significant statistical differences (Two-way ANOVA with Contrast Analysis as post hoc test, *P*<0.05).

*B. tenagophila* biomass was significantly affected by the interaction of fish and nutrient addition (*F* = 20.49, *P*<0.0001, Fig. 1 a). Higher values were observed in the N1F0 treatment, but N1F1 did not differ from the N0F0 treatment (*P*>0.05). Nutrient addition had significant positive effects on snails' C (*F* = 60.72, *P*<0.001– Fig. 1 b), N (*F* = 47.34, *P*<0.001– Fig. 1 c), and P content (*F* = 40.43, *P*<0.001– Fig. 1 d). C∶P (*F* = 9.26, *P*<0.010 – Fig. 1 f) and N∶P (*F* = 5.76, *P*<0.033 – Fig. 1 g) ratios were significantly lower with nutrient addition, but no nutrient effect was observed on the C∶N ratio (*F* = 1.16, *P*<0.623 – Fig. 1 e). No significant effect of fish presence was observed on snail N and P body content or N∶P nutrient ratio (*P*>0.05). However, *B. tenagophila* C content was significantly affected by fish presence. C content decreased with fish addition, which subsequently directly affected C∶N and C∶P ratios (Fig. 1 e, f).

#### Periphytic Total Biomass

**Figure 2:**
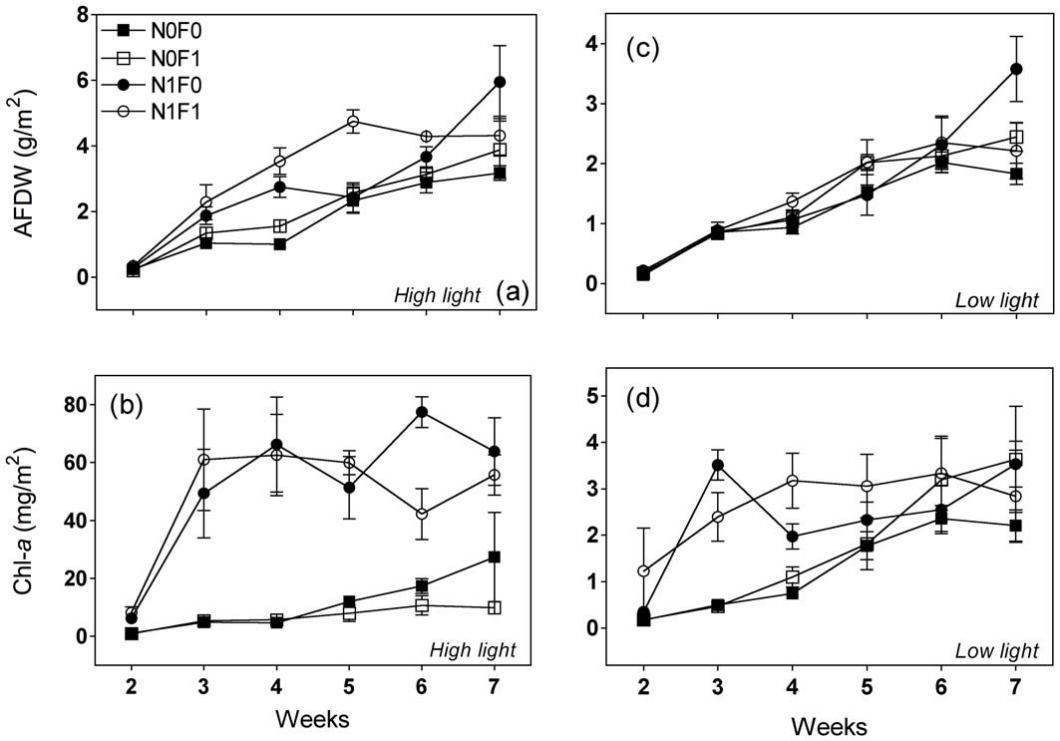
Periphyton total biomass (top – a and c) and algal biomass (bottom b and d) over the time at different light regimes (High light – a and b; Low light – c and d). Treatment abbreviations are the same as in figure 1. Circles indicate enriched treatments and unfilled symbols indicate presence of fish. Data are means ± SE. For statistical differences see the text.. Note the differences on Y-axis scales for each graph.

Nutrient addition, fish presence and light significantly affected periphytic total biomass (Fig. 2 a and c, Table S1), and strong interactions were observed among these treatments. At the fourth week, significantly higher total periphytic biomass values were observed with joint addition of nutrients and fish (Fig. 2, *F* = 5.80, *P* = 0.003). Despite the slight increase over the weeks, total periphytic biomass was significantly lower in low light conditions (Fig. 2 c, Table S1), and no significant effect was observed for nutrient addition in low light conditions except for a small effect in the last week (Fig. 2, *F* = 5.48, *P* = 0.037).

### Periphytic autotrophic biomass

Light and nutrients positively affected periphytic autotrophic biomass (Fig. 2 c and d, Table S1). Periphytic Chl-*a* values were significantly higher with nutrient addition high light conditions. Despite the strong trend, the *light × fish* interaction was not significant (*F* = 2.91, *P* = 0.113, Table S1). However, fish presence had a noticeably negative effect on periphytic autotrophic biomass in high light conditions (Fig. 2 b). This pattern was sustained by the significant interactions among *light × time × fish* (*F* = 3.22, *P* = 0.012, Table S1). At the sixth week, periphytic autotrophic biomass in the N1F1 treatment was significantly lower than in the treatment with only nutrient addition (Fig. 2 b, *F* = 4.34, *P* = 0.049). Even without nutrient addition, there was a noticeable but not significant tendency toward a negative effect of fish on periphytic autotrophic biomass (Fig. 2 b, *F* = 3.19, *P* = 0.099).

### Carbon to Chl-*a* ratio

**Figure 3:**
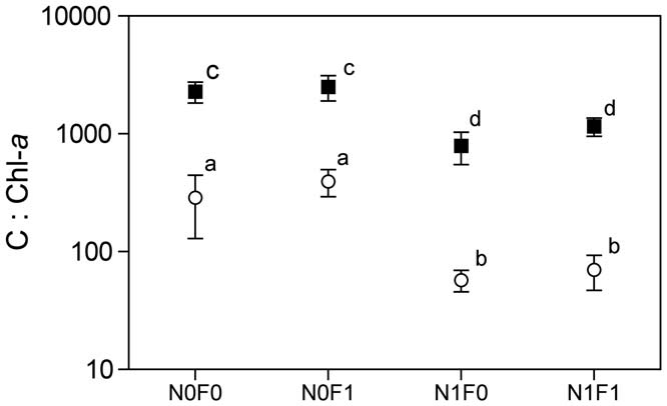
Periphyton Carbon∶Chlorophyll-a ratio in high (○) and low (▪) light conditions. Treatment abbreviations are the same as in figure 1. Each point represents averaged values of three weeks (*n* = 12) ±SE. Different letters above bars represent significant statistical differences (Two-way ANOVA with Contrast Analysis as post hoc test, *P*<0.05).

Both nutrients and light significantly affected the C∶Chl-*a* ratio in the periphytic community (Fig. 3, Table S1). Nutrient addition significantly decreased periphytic C∶Chl-*a* ratios, which reflects a greater contribution of autotrophic biomass to total periphytic biomass. The effect of light on C∶Chl-*a* was even stronger, promoting an increase in the proportion of autotrophic biomass relative to the total periphytic biomass. Despite the slight positive effect of fish presence on average C∶Chl-*a* ratios (Fig. 3), it was not statistically significant (*F* = 1.60, *P* = 0.2298, Table S1).

### Periphytic Stoichiometry

**Figure 4:**
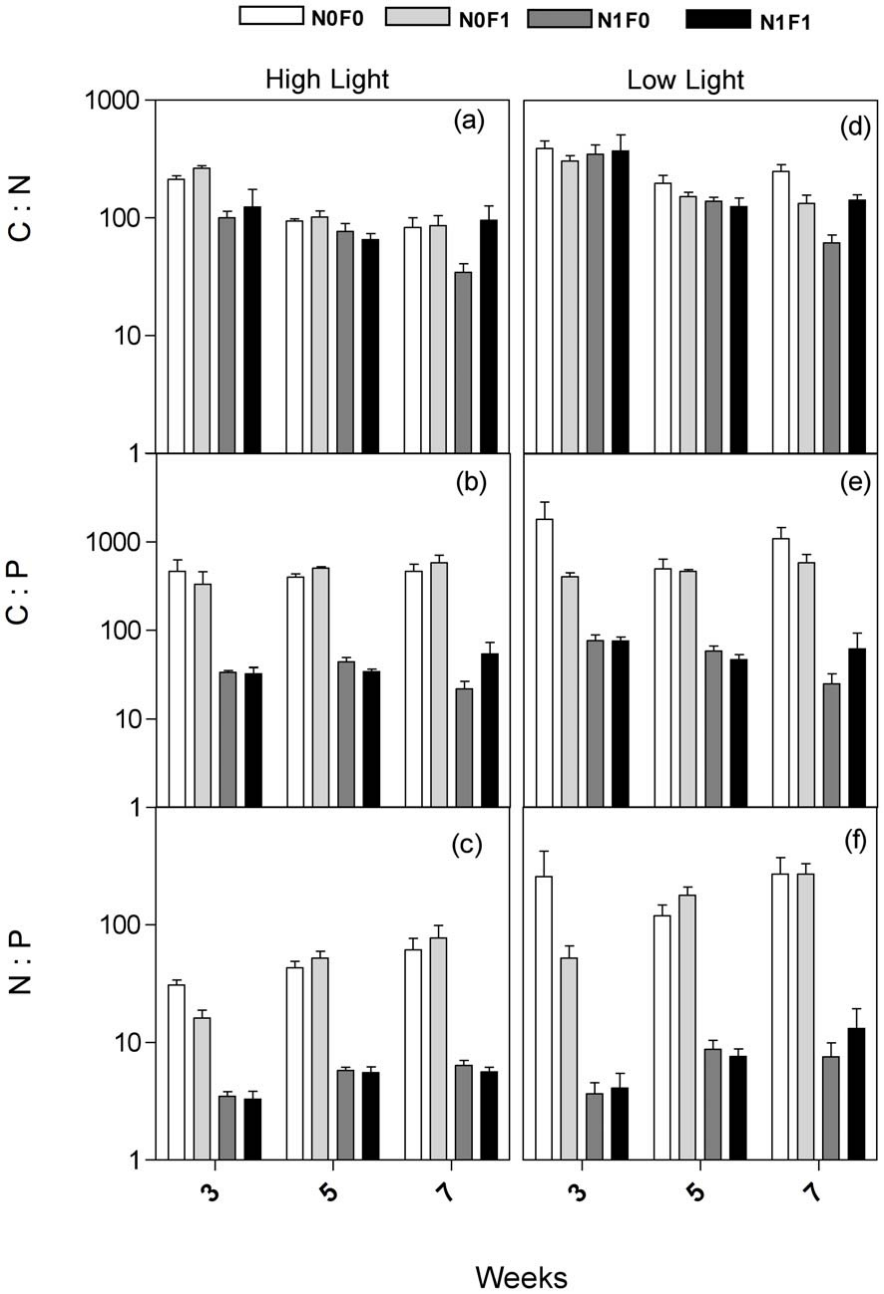
Response of C∶N∶P stoichiometric ratios in periphyton biomass to different light regimes (high light – a, b, c; low light – d, e, f) and treatments. Treatment abbreviations are the same as in figure 1. Each bar represents mean values for each week (*n* = 4) +1SE.

Periphytic C∶P and N∶P ratios drastically decreased in the treatments that received nutrient addition (Fig. 4 b, c, e and f, Table S2). C∶N ratios also decreased with nutrient addition, but the differences among treatments were much lower compared to the C∶P or N∶P ratios. Fish had a transient effect on periphyton stoichiometry, and this effect was dependent on light and nutrient availability, viewed by the significant interactions of *time × fish × nutrient* for C∶N and N∶P ratios (*F* = 4.65, *P* = 0.0229 and *F* = 5.18, *P* = 0.0134, respectively, Table S2) and *light×time×fish* for C∶N ratio (*F* = 5.13, *P* = 0.0446, Table S2). Average changes in periphyton stoichiometry over the course of the experiment were observed for both C∶N and N∶P ratios (Table S2 – Figure 4). C∶N ratios presented a slight decrease throughout the weeks when compared with the third week (Figure 4 a and d). However, N∶P ratios presented a slight increase over time (Figure 4 c and f). Light availability also had a significant overall effect on periphytic stoichiometry (Table S2). Both C∶N and C∶P ratios were slightly greater in low light conditions, but N∶P ratios presented slightly lower values.

## Discussion

We hypothesized that the addition of fish would positively affect periphyton biomass, and the strength of such effect would depend on resource availability. The direction and magnitude of the effect of fish was dependent on both nutrient and light availability; however, the direction of the effect was depended also on the fraction of periphytic biomass (i.e., total or autotrophic), which only partially supports our first hypothesis. We observed that periphytic C∶N and C∶P ratios increased with the reduction of light, contradicting our second hypothesis hypothesis. Nevertheless, our results also showed that benthic consumers' nutrient content increased as a response to periphyton nutrient enrichment, supporting our third hypothesis and highlighting consumers' body nutrient plasticity and the propagation of a stoichiometric that cascades up the food web.

The balance between light and nutrients is critical for benthic communities because restrictions of light availability may limit the propagation of nutrient effects [35]. This assumes a greater importance in brown-water lakes, where the high concentration of dissolved carbon leads to the attenuation of light in the water column, strongly affecting the productivity of benthic communities [36]. It has been suggested that a substantial part of the photosynthesis in clear-water lakes occurs in benthic algal communities, and the fraction of benthic productivity that is transferred to consumers decreases as the amount of dissolved carbon increases in the water column [14]. Consequently, changes in light abundances due to dissolved colored carbon can weaken the strength of trophic interactions by limiting the productivity of basal trophic levels [37], as we observed even in high nutrient conditions. This result highlights that mild nutrient loads that can increase the relative importance of predators' indirect effects on basal trophic levels [10] may be diminished in dark-water lakes. Additionally, our experimental design allowed for the vertical migration of herbivores, greater biomass and lower C∶N∶P ratios of the periphyton located at the surface (i.e., high light condition) of the experimental enclosures that may have influenced the numerical and functional responses of herbivores in this region. In general, herbivores increase their herbivory rates in conditions where food is abundant [38] and increase their numerical response through dispersion rather than reproduction, actively selecting patches of greater food quantity or quality [39]. Consequently, in addition to the indirect effect of fish predation on the periphyton community, a greater importance of consumer control is expected at the surface.

The degree of omnivory may increase with a decrease of optimal prey types, such as in conditions of low productivity, which may subsequently affect the strength of trophic cascades. The lower periphytic biomass in N0F1 treatment and observed overall negative effect of fish on periphytic autotrophic biomass may be a result of direct biomass consumption by fish. Gelwick and Matthews [40] have shown that fish can selectively feed on periphyton, exerting greater herbivory on more prostrated algae forms. This was confirmed by our personal observations. By directly feeding on loosely adnate algae, fish negatively affected periphytic autotrophic biomass content, resulting in weaker trophic cascades. Alternatively, treatments that received nutrient addition supported a greater biomass of qualitatively superior food items (i.e., zooplankton and gastropods), increasing fish carnivory and reducing food web connectivity [41], allowing a stronger cascade effect [42]. However, the positive effect of fish on phytoplankton biomass through zooplankton predation can negatively affect periphyton autotrophic biomass by reducing the light availability to the periphytic community [43], as observed here through the significant reduction of Secchi disk depth in the N1F1 treatment. Thus, despite the positive and greater effect of predators on the base of the food web in conditions of higher productivity (i.e., more nutrients and light), the omnivory behavior may have weakened predators' net effect on periphyton biomass through indirect pathways, particularly by the reduction of light availability to the benthic primary producer.

We observed that nutrient addition drastically reduced periphyton C∶P and N∶P ratios, suggesting strong phosphorus limitation experienced by the periphyton community. However, light availability significantly affected periphytic C∶N and C∶P ratios contrary to the expected effects based on the light∶nutrient hypothesis. We observed that molar C∶P ratios were significantly higher in low light conditions in contrast with recent findings regarding periphyton stoichiometry along light gradients [28]. We believe that this mismatch may be a result of the small contribution of autotrophic biomass to total periphytic biomass in low light conditions. Shifts in community composition along resource gradients have been predicted by experimental and theoretical works [44]. These shifts, especially for algal composition, correlate with variations in the nutrient stoichiometry of the entire algal assemblage in some circumstances, highlighting the occurrence of taxa–stoichiometry correlations [25]. However, links between periphyton biomass composition and the light∶nutrient hypothesis remain largely unexplored. We believe that the content variation in periphyton C∶N∶P ratios due to changes in algal physiology might be buffered by the overwhelming contribution of non-autotrophic biomass to the periphyton community with reduced light availability. Additionally, autotrophic and non-autotrophic (e.g., bacteria) biomass within periphyton vary in their nutrient content [45], which may drive changes in the nutrient stoichiometry of the entire assemblage along resource gradients, as these gradients modify the periphytic biomass composition. Bacteria may be an important component of periphyton non-autotrophic biomass [46], driving changes in N∶P stoichiometry in low light conditions when they directly assimilate inorganic nutrients from the water column and incorporate them into their biomass. Another important component that must be considered to explain the variation in periphyton C∶N∶P ratios among light conditions is the effect of grazers on periphyton stoichiometry. The unselective grazing of benthic invertebrates reduces the proportion of detritus, they remove algae and detritus, but only algae regenerate [22]. Therefore, grazing increases the proportion of live organisms compared with detritus. The great majority of benthic grazers were located at the upper part of the enclosures wall (i.e., in high light conditions). By reducing the proportion of C-rich but nutrient-poor detritus or even altering biomass turnover rates, grazers may increase the relative nutrient content of the remaining periphyton, leading to lower periphyton C∶nutrients ratios. This mechanism is especially important because it highlights the importance of herbivores to the relationship between light and periphyton nutrient content. Hall et al. [47] found for pelagic communities that algae nutrient∶C ratios were correlated with light∶P ratios only when consumers were present. Our results suggest that habitat connection and heterogeneity, which in this specific case can lead to differential resource use by gastropods across the depth-related biotope space [48], which encompass the natural heterogeneity along the water column vertical axis, can be a proximate mechanism explaining the relationship between light and benthic producers' stoichiometry.

Despite the strong effects of fish predation and resources on periphytic biomass, our results suggest an asymmetry in the strength of fish and resource effects on periphyton stoichiometry. Nutrients and light strongly affected periphyton stoichiometry, but only small effects of fish presence were observed. Fish can positively affect lower trophic levels through distinct mechanisms, such as nutrient recycling or by reducing herbivory-induced mortality rates [49]. In the present study, we believe that the positive effect of fish on periphytic biomass can be attributed mainly to the trophic cascade effect because no significant difference in water-column nutrient concentrations was observed with the addition of fish. Thus, the effect of fish on nutrient regeneration may have played a minor role in the present experiment, resulting in the small effect of fish presence on periphyton stoichiometry. However, predation should be linked to periphyton stoichiometry patterns through the reduction of grazers' density [50]. The slight positive effect of fish presence on periphytic C∶N ratios may be a result of fish predation on herbivores, reducing the grazing pressure on periphyton and potentially diminishing periphyton biomass turnover and nutrient uptake [51].

The body nutrient content (N and P) of gastropods significantly increased with nutrient addition, shown by a reduction of gastropod C∶P and N∶P nutrient ratios. Because these animals are not able to directly assimilate inorganic nutrients into their biomass, we conclude that such modification was driven by changes in the stoichiometry of their food (i.e., periphyton). Food with low P-content has tended to reduce grazers' P content, suggesting that consumers' high C∶nutrients stoichiometric ratios may be a result of physiological limitations rather than having relatively low and fixed nutrient requirements [23], [52]. Our results reflect this pattern because those treatments with lower periphyton C∶N∶P ratios also presented richer gastropod nutrient content in dry weight, evidencing the co-variance of periphyton-grazer stoichiometry to some degree. An alternative explanation for *B. tenagophila* body nutrient flexibility is that grazers might have grown faster in enriched treatments due to higher periphytic biomass, producing more RNA, which leads to higher P content [53]. However, this mechanism does not explain the increase in grazers' N content observed in our experiment. The analysis of *B. tenagophila* C content between treatments highlights another aspect of how resource limitation can affect consumers' stoichiometry. The positive effect of nutrient addition on *B. tenagophila* C content may reflect the suppression of energy limitation experienced by the gastropod population in low resource conditions (i.e., low periphyton biomass), a hypothesis that is corroborated by the significant negative effect of fish presence on *B. tenagophila* C content. Many studies have shown that predation risk can affect prey in a variety of ways, especially by reducing its foraging activity [54], which can lead to individual starvation [55]. These results suggest that differences in gastropods' stoichiometry can result from physiological constraints (e.g., by feeding on P-poor periphyton) observed in primary producers in conditions of low nutrient supply and suggested by other recent studies [21], highlighting the fact that nutritional limitation can travels up the food web. Therefore, furthers studies must take in account the flexibility of the nutrient content of grazer body tissues and possibly the propagation of stoichiometric cascades through the food web.

Recent studies have suggested that light attenuation due to colored dissolved carbon in the water column of humic lakes could strongly reduce the productivity of benthic primary producers, ultimately affecting the productivity of higher trophic levels [14]. However as observed in the present, experiment light limitation can also increase the C∶ nutrient ratios of benthic primary producers. Because benthic consumers' body stoichiometry is constrained by nutrient limitation, we believe that light attenuation negatively affects higher trophic levels' productivity not just by reducing primary productivity but also by increasing the mineral limitation of herbivore growth. However, is not clear if mineral limitation of herbivore growth is widespread in the literature. Hill et al [16] found no effect of food quality on snail growth, which was attributed to competition for limited food due to low predation or other mortality factors, although such effect have been observed for pelagic [56] and even benthic [57] organisms in previous studies. Our results showed that average consumers nutrient N and P content was slight higher in the treatments with fish and nutrients addition, which corroborates with Hill et al [16] findings, however these differences were not statistically significant. Despite the positive effect of predation on the likelihood of consumer mineral limitation, the lack of an effect of food quality on consumers' growth can be specially related to consumer growth rates, because slow-growing herbivores theoretically can tolerate food with relatively low N and P content [16]. Although growth rate is an intrinsic feature of each species, many freshwater gastropods grow in relation with temperature, and the growth rate is relatively high for the genus *Biomphalaria* compared with other groups [58]. Thus, at least for systems with similar characteristics to the one in the present study or even other warm water lakes, light attenuation may affect benthic consumer productivity through reduced primary productivity and nutrient limitation.

Considering the habitat preference of small omnivorous fish such as *H. bifasciatus* and its often high abundance in tropical shallow lakes and relative tolerance to turbid water [29], it is likely that these species promote strong modifications in the littoral regions of these lakes. However, especially in humic lakes where light attenuation through the water column is in generally severe, this effect could be diminished or at least constrained to the top layers of the ecosystem. As observed in the present experiment, light attenuation throughout the water column may affect periphyton biomass and stoichiometry and the degree to which nutrients and predators affect them. Our results also suggest that further development of the light∶nutrient hypothesis applied to benthic habitats could integrate changes in periphyton biomass composition over grazer depth-related biotope space and gradients of resource supply, especially light. In addition to the acknowledged stoichiometry plasticity of herbivore consumers, such a synthesis should yield a better understanding of the energy transference through benthic food webs, which significantly supports top-predators biomass in many lakes worldwide [59].

## Supporting Information

Figure S1 Schematic representation of the experimental design. Treatments are represented as: N0F0 (no fish or nutrient addition); N0F1 (only fish addition); N1F0 (only nutrient addition); N1F1 (both fish and nutrient addition). The sizes of the elements in the scheme are not drawn to scale. See the section Methods: *Experimental design and Setup* for more details.
(TIFF)

Figure S2 Photographs of an experimental enclosure (N1F0 treatment). The larger photograph shows the freshwater snails *Biomphalaria tenagophila* as the predominantly specie colonizing the upper part of the enclosure wall. The smaller photograph is a close-up of high densities of snails. Photos Credit: A. Caliman.
(TIFF)

Table S1 Results of the univariate factorial Within-Subject ANOVA for periphyton AFDW, chlorophyll-a (Chl-*a*) and Chl-*a*/AFDW ratio. The *F* ratio and *P*-values for all main factors and their interactions are presented in the table. *P*-values for Within-Subjects were corrected by the Huynh-Feldt adjustment. Bolded *P*-values highlight significant treatment effects (*P*<0.05).
(DOC)

Table S2 Results of the univariate factorial Within-Subject ANOVA for periphyton stoichiometry (C∶N; C∶P and N∶P ratios). The *F* ratio and *P*-values for all main factors and their interactions are presented in the table. *P*-values for Within-Subjects were corrected by the Huynh-Feldt adjustment. Bolded *P*-values highlight significant treatment effects (*P*<0.05).
(DOC)

## References

[1] Gruner DS, Smith JE, Seabloom EW, Sandin Sa, Ngai JT (2008). A cross-system synthesis of consumer and nutrient resource control on producer biomass.. Ecology letters.

[2] Power ME (1992). Top-down and bottom-up forces in food webs: do plants have primacy.. Ecology.

[3] Vadeboncoeur Y, Vander-Zanden MJ, Lodge DM (2002). Putting the lake back together: reintegrating benthic pathways into lake food web models.. Bioscience.

[4] Brönmark C (1994). Effects of tench and perch on interactions in a freshwater, benthic food chain.. Ecology.

[5] McCollum EW, Crowder LB, McCollum SA (1998). Complex interactions of fish, snails, and littoral zone periphyton.. Ecology.

[6] McIntosh AR, Townsend CR (1996). Interactions between fish, grazing invertebrates and algae in a New Zealand stream: a trophic cascade mediated by fish-induced changes to grazer behaviour?. Oecologia.

[7] Bertrand KN, Gido KB (2007). Effects of the Herbivorous Minnow, Southern Redbelly Dace (Phoxinus erythrogaster), on Stream Productivity and Ecosystem Structure.. Oecologia.

[8] Chase JM (2003). Strong and weak trophic cascades along a productivity gradient.. Oikos.

[9] Brett MT, Goldman CR (1996). A meta-analysis of the freshwater trophic cascade..

[10] Flecker AS, Taylor BW, Bernhardt ES, Jm (2002). Interactions between herbivorous fishes and limiting nutrients in a tropical stream ecosystem.. Ecology.

[11] Schindler DE, Scheuerell MD (2002). Habitat coupling in lake ecosystems.. Oikos.

[12] Vadeboncoeur Y, McCann KS, Vander Zanden MJ, Rasmussen JB (2005). Effects of multi-chain omnivory on the strength of trophic control in lakes.. Ecosystems.

[13] Vadeboncoeur Y, Peterson G, Vander Zanden MJ, Kalff J (2008). Benthic algal production across lake size gradients: interactions among morphometry, nutrients, and light.. Ecology.

[14] Karlsson J, Bystrom P, Ask J, Ask P, Persson L (2009). Light limitation of nutrient-poor lake ecosystems.. Nature.

[15] Qin P, Mayer CM, Schulz KL, Ji X, Ritchie ME (2007). Ecological stoichiometry in benthic food webs: Effect of light and nutrients on periphyton food quantity and quality in lakes.. Limnology and Oceanography.

[16] Hill WR, Smith JG, Stewart AJ (2010). Light, nutrients, and herbivore growth in oligotrophic streams.. Ecology.

[17] Sanches L, Guariento RD, Caliman A, Bozelli RL, Esteves FA (2011). Effects of nutrients and light on periphytic biomass and nutrient stoichiometry in a tropical black-water aquatic ecosystem.. Hydrobiologia.

[18] Fanta SE, Hill WR, Smith TB, Roberts BJ (2010). Applying the light: nutrient hypothesis to stream periphyton.. Freshwater Biology.

[19] Dickman EM, Newell JM, Gonzalez MJ, Vanni MJ (2008). Light, nutrients, and food-chain length constrain planktonic energy transfer efficiency across multiple trophic levels.. Proceedings of the National Academy of Sciences of the United States of America.

[20] Quigg A, Finkel ZV, Irwin AJ, Rosenthal Y, Ho TY (2003). The evolutionary inheritance of elemental stoichiometry in marine phytoplankton.. Nature.

[21] Boersma M, Aberle N, Hantzsche FM, Schoo KL, Wiltshire KH (2008). Nutritional Limitation Travels up the Food Chain.. International Review of Hydrobiology.

[22] Hillebrand H, Frost PC, Liess A (2008). Ecological stoichiometry of indirect grazer effects on periphyton nutrient content.. Oecologia.

[23] Cross WF, Benstead JP, Rosemond AD, Wallace JB (2003). Consumer-resource stoichiometry in detritus-based streams.. Ecology Letters.

[24] Elser JJ, Urabe J (1999). The stoichiometry of consumer-driven nutrient recycling: theory, observations, and consequences.. Ecology.

[25] Hall SR, Shurin JB, Diehl S, Nisbet RM (2007). Food quality, nutrient limitation of secondary production, and the strength of trophic cascades.. Oikos.

[26] Esteves FA, Caliman A, Santangelo JM, Guariento RD, Farjalla VF (2008). Neotropical Coastal Lagoons: An appraisal of their Biodiversity, Functioning, Threats and Conservation Management.. Brazilian Journal of Biology.

[27] Thiengo SC, Fernandez MA, Boaventura MF, Santos SB, Mattos AC (2002). Freshwater Snails and Schistosomiasis Mansoni in the State of Rio de Janeiro, Brazil: II - Centro Fluminense Mesoregion.. Memorial Instituto Oswaldo Cruz.

[28] Danger M, Lacroix G, Oumarou C, Benest D, Mériguet J (2008). Effects of food-web structure on periphyton stoichiometry in eutrophic lakes: a mesocosm study.. Freshwater Biology.

[29] Sánchez-Botero JI, Caramaschi EP, Garcez DS (2008). Spatio-temporal variation in fish assemblage in a coastal lagoon without direct contact with the sea (southeastern Brazil).. Journal of Coast Research.

[30] Carvalho TP, Bertaco VA (2006). Two new species of Hyphessobrycon (Teleostei: Characidae) from the upper rio Tapajos basin on Chapada dos Perecis, Central Brazil.. Neotropical Ichthyology.

[31] APHA (1989). Standard methods for examination of water and wastewater.

[32] Solorzano L (1969). Determination of ammonia in natural waters by phenolhypochlorite method.. Limnology and Oceanography.

[33] Mackereth FJH, Heron J, Talling JF (1978). Water analysis: some revised methods for limnologists.

[34] Hargrave CW (2006). A test of three alternative pathways for consumer regulation of primary productivity.. Oecologia.

[35] Eriksson BK, Rubach A, Hillebrand H (2006). Biotic habitat complexity controls species diversity and nutrient effects on net biomass production.. Ecology.

[36] Ask J, Karlsson J, Lennart P, Ask P, Bystrom P (2009). Terrestrial organic matter and light penetration: Effects on bacterial and primary production in lakes.. Limnology and Ocenography.

[37] Darcy-Hall TL (2006). Relative strengths of benthic algal nutrient and grazer limitation along a lake productivity gradient.. Oecologia.

[38] Sommer U (1999). The impact of herbivore type and grazing pressure on benthic microalgal diversity.. Ecology Letters.

[39] Cruz-Rivera E, Hay ME (2000). Can quantity replace quality? Food choice, compensatory feeding, and fitness of marine mesograzers.. Ecology.

[40] Gelwick FP, Matthews WJ (1992). Effects of an algivorous minnow on temperate stream ecosystem properties.. Ecology.

[41] Guariento R, Carneiro L, Caliman A, Bozelli R, Fonseca LJ (2010). Interactive effects of omnivorous fish and nutrient loading on net productivity regulation of phytoplankton and periphyton.. Aquatic Biology.

[42] Bascompte J, Melian CJ, Sala E (2005). Interaction strength combinations and the overfishing of a marine food web.. Proceedings of the National Academy of Sciences of the United States of America.

[43] McCollum EW, Crowder LB, McCollum SA (1998). Complex interactions of fish, snails, and littoral zone periphyton.. Ecology.

[44] Passarge J, Hol S, Escher M, Huisman J (2006). Competition for nutrients and light: Stable coexistence, alternative stable states, or competitive exclusion?. Ecological Monographs.

[45] Fagerbakke KM, Heldal M, Norland S (1996). Content of carbon, nitrogen, oxygen, sulfur and phosphorus in native aquatic and cultured bacteria.. Aquatic Microbial Ecology.

[46] Carr GM, Morin A, Chambers PA (2005). Bacteria and algae in stream periphyton along a nutrient gradient.. Freshwater Biology.

[47] Hall SR, Leibold Ma, Lytle Da, Smith VH (2007). Grazers, producer stoichiometry, and the light ∶ nutrient hypothesis revisited.. Ecology.

[48] Hutchinson GE (1978). An Introduction to Population Ecology.

[49] Vanni MJ, Layne CD, Arnott SE (1997). “Top-down” trophic interactions in lakes: Effects of fish on nutrient dynamics.. Ecology.

[50] Liess A, Hillebrand H (2006). Grazer-periphyton interactions: reciprocal influences of periphyton and grazer nutrient stoichiometry.. Journal of the North American Benthological Society.

[51] Hillebrand H, De Montpellier G, Liess A (2004). Effects of macrograzers and light on periphyton stoichiometry.. Oikos.

[52] Fink P, Elert EV (2006). Physiological responses to stoichiometric constraints: nutrient limitation and compensatory feeding in a freshwater snail.. Oikos.

[53] Liess A, Hillebrand H (2006). Role of nutrient supply in grazer-periphyton interactions: reciprocal influences of periphyton and grazer nutrient stoichiometry.. Journal of the North American Benthological Society.

[54] Preisser EL, Bolnick DI (2008). The Many Faces of Fear: Comparing the Pathways and Impacts of Nonconsumptive Predator Effects on Prey Populations.. Plos One.

[55] Abrams PA (2007). Defining and measuring the impact of dynamic traits on interspecific interactions.. Ecology.

[56] Hessen DO, Færøvig PJ, Andersen T (2002). Light, nutrients, and P:C ratios in algae: grazer performance related to food quality and quantity.. Ecology.

[57] Stelzer RS, Lamberti GA (2002). Ecological stoichiometry in running waters: periphyton chemical composition and snail growth.. Ecology.

[58] Nunez V (2010). Differences on allocation of available resources, in growth, reproduction, and survival, in an exotic gastropod of Physidae compared to an endemic one.. Iheringia Sér Zoologia.

[59] Vadeboncoeur Y, Jeppesen E, Zanden VMj, Schierup HH (2003). From Greenland to green lakes: cultural eutrophication and the loss of benthic energy pathways in lakes.. Limnology and Oceanography.

