# Supplementary figures and images for: Food Web Architecture and Basal Resources Interact to Determine Biomass and Stoichiometric Cascades along a Benthic Food Web
Source: PLoS One. 2011 Jul 18;6(7):e22205. doi: 10.1371/journal.pone.0022205 (PMC3138757; doi:10.1371/journal.pone.0022205)

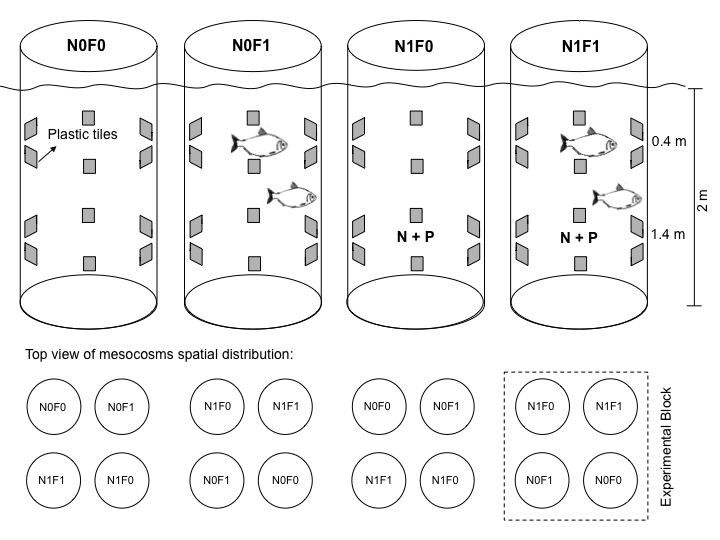

Supplement: Figure S1 — Schematic representation of the experimental design. Treatments are represented as: N0F0 (no fish or nutrient addition); N0F1 (only fish addition); N1F0 (only nutrient addition); N1F1 (both fish and nutrient addition). The sizes of the elements in the scheme are not drawn to scale. See the section Methods: Experimental design and Setup for more details. (TIFF) [file pone.0022205.s001.tiff]

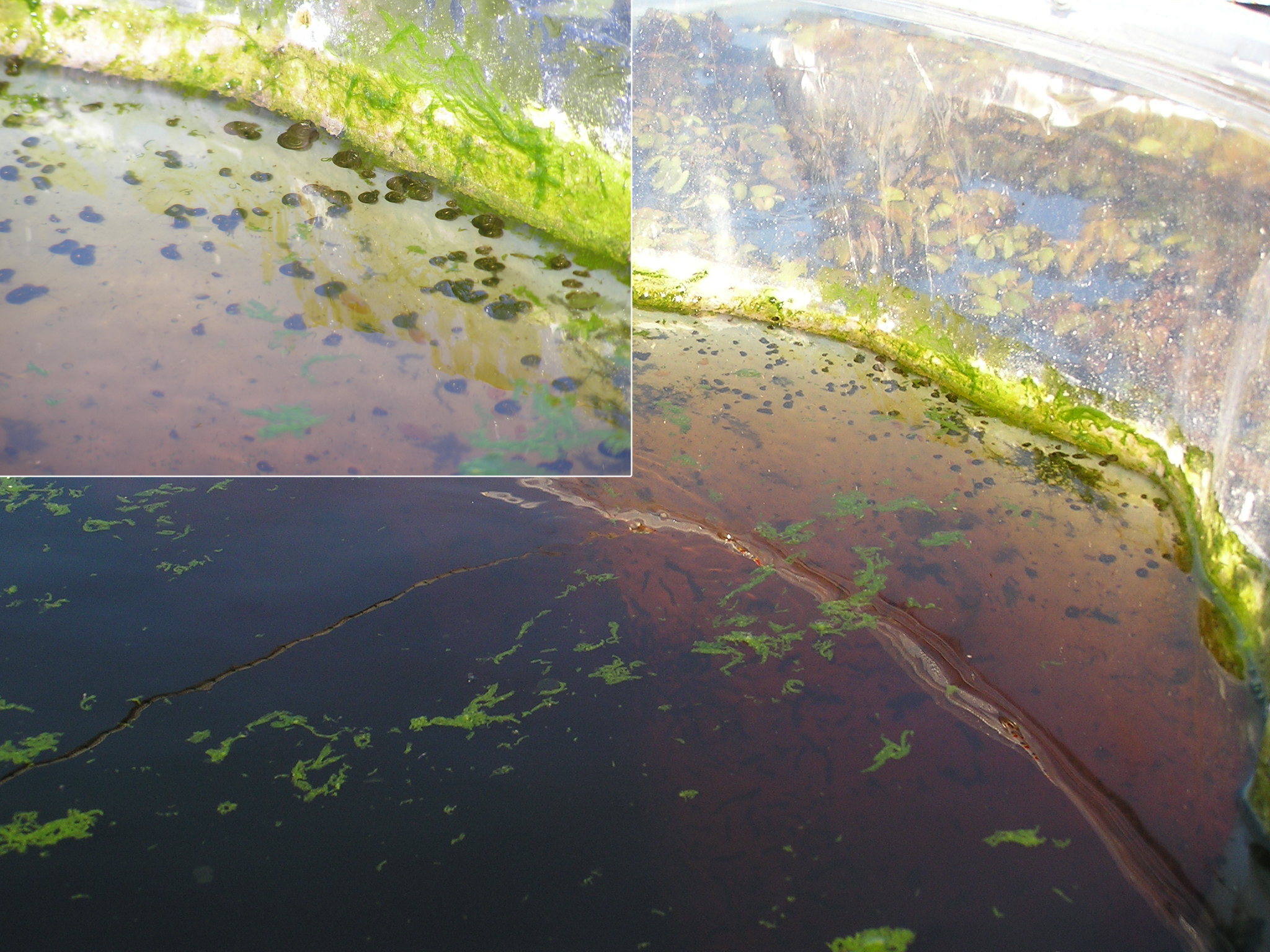

Supplement: Figure S2 — Photographs of an experimental enclosure (N1F0 treatment). The larger photograph shows the freshwater snails Biomphalaria tenagophila as the predominantly specie colonizing the upper part of the enclosure wall. The smaller photograph is a close-up of high densities of snails. Photos Credit: A. Caliman. (TIFF) [file pone.0022205.s002.tiff]
