# Supplementary material for: Food Web Architecture and Basal Resources Interact to Determine Biomass and Stoichiometric Cascades along a Benthic Food Web
Source: PLoS One. 2011 Jul 18;6(7):e22205. doi: 10.1371/journal.pone.0022205 (PMC3138757; doi:10.1371/journal.pone.0022205)
Supplement: Table S1 — Results of the univariate factorial Within-Subject ANOVA for periphyton AFDW, chlorophyll-a (Chl-a) and Chl-a/AFDW ratio. The F ratio and P-values for all main factors and their interactions are presented in the table. P-values for Within-Subjects were corrected by the Huynh-Feldt adjustment. Bolded P-values highlight significant treatment effects (P<0.05). (DOC) [file pone.0022205.s003.doc]

| Factor | AFDW | |  | Chl-*a* | |  | C:Chl-*a* | |
| --- | --- | --- | --- | --- | --- | --- | --- | --- |
|  | *F* | *P-value* |  | *F* | *P-value* |  | *F* | *P-value* |
| Fish (F) | **4.97** | **0.0455** |  | 0.132 | 0.7225 |  | 1.60 | 0.2298 |
| Nutrient (N) | **24.50** | **0.0003** |  | **49.76** | **<0.0001** |  | **30.13** | **0.0001** |
| F x N | 0.14 | 0.7061 |  | 0.04 | 0.8297 |  | 0,05 | 0.9474 |
| Light (L) | **82.97** | **<0.0001** |  | **547.83** | **<0.0001** |  | **347.95** | **<0.0001** |
| L x F | 1.10 | 0.3138 |  | 2.91 | 0.1132 |  | 4.098 | 0.065 |
| L x N | **10.91** | **0.0063** |  | **30.88** | **0.0001** |  | **20.299** | **0.0007** |
| L x F x N | 0.54 | 0.4763 |  | 0.21 | 0.6484 |  | 0.04 | 0.8279 |
| Time (T) | **285.90** | **<0.0001** |  | **121.22** | **<0.0001** |  | 1.40 | 0.2358 |
| T x F | **4.645** | **0.0011** |  | 0.84 | 0.5260 |  | 1.21 | 0.3138 |
| T x N | **3.588** | **0.0066** |  | **8.77** | **<0.0001** |  | **7.55** | **<0.001** |
| T x F x N | **4.779** | **0.0009** |  | 0.71 | 0.6143 |  | 0.34 | 0.8815 |
| L x T | **7.06** | **<0.0001** |  | 0.02 | 0.2602 |  | 0.232 | 0.9468 |
| L x T x F | 0.37 | 0.8672 |  | **3.224** | **0.0120** |  | 1.15 | 0.3439 |
| L x T x N | **3.597** | **0.0065** |  | **4.023** | **0.0032** |  | **4.00** | **0.0033** |
| L x T x F x N | 0.95 | 0.4548 |  | 2.22 | 0.0635 |  | 1.19 | 0.3231 |
