# Supplementary material for: Food Web Architecture and Basal Resources Interact to Determine Biomass and Stoichiometric Cascades along a Benthic Food Web
Source: PLoS One. 2011 Jul 18;6(7):e22205. doi: 10.1371/journal.pone.0022205 (PMC3138757; doi:10.1371/journal.pone.0022205)
Supplement: Table S2 — Results of the univariate factorial Within-Subject ANOVA for periphyton stoichiometry (C∶N; C∶P and N∶P ratios). The F ratio and P-values for all main factors and their interactions are presented in the table. P-values for Within-Subjects were corrected by the Huynh-Feldt adjustment. Bolded P-values highlight significant treatment effects (P<0.05). (DOC) [file pone.0022205.s004.doc]

| Factor | C:N | |  | C:P | |  | N:P | |
| --- | --- | --- | --- | --- | --- | --- | --- | --- |
|  | *F* | *P-value* |  | *F* | *P-value* |  | *F* | *P-value* |
| Fish (F) | 1.66 | 0.2236 |  | 0.83 | 0.3784 |  | 0.09 | 0.7611 |
| Nutrient (N) | **28.42** | **0.0002** |  | **751.58** | **>0.0001** |  | **81.65** | **>0.001** |
| F x N | 1.76 | 0.2113 |  | 0.84 | 0.3749 |  | 0.08 | 0.7799 |
| Light (L) | **47.01** | **>0.001** |  | **13.01** | **0.0036** |  | **14.18** | **0.0044** |
| L x F | 2.68 | 0.0906 |  | 0.04 | 0.8416 |  | 0.02 | 0.8888 |
| L x N | 0.01 | 0.9307 |  | 0.09 | 0.9654 |  | **11.41** | **0.0081** |
| L x F x N | 1.78 | 0.2083 |  | 0.70 | 0.4175 |  | 0.08 | 0.7722 |
| Time (T) | **50.81** | **>0.001** |  | 1.28 | 0.3031 |  | **10.83** | **0.0081** |
| T x F | 0.43 | 0.6538 |  | 4.37 | 0.0283 |  | 1.39 | 0.2723 |
| T x N | 1.55 | 0.2333 |  | 0.20 | 0.8148 |  | **8.24** | **0.0028** |
| T x F x N | **4.65** | **0.0229** |  | 1.37 | 0.2730 |  | **5.18** | **0.0134** |
| L x T | 2.01 | 0.0927 |  | **4.49** | **0.0220** |  | **8.39** | **0.0026** |
| L x T x F | **5.13** | **0.0446** |  | 0.12 | 0.8814 |  | 0.34 | 0.7095 |
| L x T x N | 2.67 | 0.0913 |  | 1.41 | 0.2693 |  | **7.79** | **0.0036** |
| L x T x F x N | 1.18 | 0.3256 |  | 0.17 | 0.8384 |  | 0.61 | 0.5516 |
